# Nerve growth factor promote osteogenic differentiation of dental pulp stem cells through MEK/ERK signalling pathways

**DOI:** 10.1111/jcmm.18143

**Published:** 2024-02-09

**Authors:** Chen Cheng, Shuai Tang, Shuyue Cui, Tong Yang, Lan Li, Mingrui Zhai, Fulan Wei, Gang Ding

**Affiliations:** ^1^ School of Stomatology Shandong Second Medical University Weifang China; ^2^ Department of Orthodontics, School and Hospital of Stomatology, Cheeloo College of Medicine Shandong University & Shandong Key Laboratory of Oral Tissue Regeneration & Shandong Engineering Laboratory for Dental Materials and Oral Tissue Regeneration & Shandong Provincial Clinical Research Center for Oral Diseases China; ^3^ Department of Stomatology Heze Municipal Hospital China

**Keywords:** dental pulp stem cells, differentiation, MEK/ERK signalling, nerve growth factor, tropomyosin receptor kinase A

## Abstract

Nerve growth factor (NGF) and its receptor, tropomyosin receptor kinase A (TrkA), are known to play important roles in the immune and nervous system. However, the effects of NGF on the osteogenic differentiation of dental pulp stem cells (DPSCs) remain unclear. This study aimed to investigate the role of NGF on the osteogenic differentiation of DPSCs in vitro and the underlying mechanisms. DPSCs were cultured in osteogenic differentiation medium containing NGF (50 ng/mL) for 7 days. Then osteogenic‐related genes and protein markers were analysed using qRT‐PCR and Western blot, respectively. Furthermore, addition of NGF inhibitor and small interfering RNA (siRNA) transfection experiments were used to elucidate the molecular signalling pathway responsible for the process. NGF increased osteogenic differentiation of DPSCs significantly compared with DPSCs cultured in an osteogenic‐inducing medium. The NGF inhibitor Ro 08‐2750 (10 μM) and siRNA‐mediated gene silencing of NGF receptor, TrkA and ERK signalling pathways inhibitor U0126 (10 μM) suppressed osteogenic‐related genes and protein markers on DPSCs. Furthermore, our data revealed that NGF‐upregulated osteogenic differentiation of DPSCs may be associated with the activation of MEK/ERK signalling pathways via TrkA. Collectively, NGF was capable of promoting osteogenic differentiation of DPSCs through MEK/ERK signalling pathways, which may enhance the DPSCs‐mediated bone tissue regeneration.

## 1 INTRODUCTION

Many diseases can cause skeletal defects in the maxillofacial region, such as trauma, inflammation and tumours. Current treatments are insufficient to repair large skeletal defects. However, higher osteogenesis occurs in engineered, vascularized and neurotized bone tissue with implanted sensory nerve and vascular bundle. Additionally, the use of vascularized iliac flaps with simultaneous neurorrhaphy between the ilioinguinal nerve and inferior alveolar nerve can effectively prevent bone resorption in mandible reconstruction and enhance dental implant quality.^1^, ^2^, ^3^

Bone tissue is highly vascularized and innervated, with close interaction between blood vessels and nerve fibres, which mutually support bone development and fracture healing.^4^, ^5^ Blood vessels transport cells to the bone surface and also supply oxygen, nutrients and hormones.^6^ The nervous system in bone tissue also influences bone cell function and actively participates in bone resorption/formation.^7^ Importantly, nerves enter bones alongside arteries and remain closely associated with blood vessels inside bone, sharing common genetic pathways and stimuli.^8^ Bone metabolism is regulated by signals generated by intrabony nerves, and these signals mediate bone mass and maintain the macro‐architecture of bone through regulating bone deposition by osteoblasts and resorption by osteoclasts.^9^ Nerve fibres are frequently identified in the trabecular bone, periosteum and the callus that forms around the ends of broken bone after bone fracture, regulating bone metabolism via secretion of neurotransmitters, neuropeptides, neurotrophins, neuronal guidance factors and participation of nerve‐resident cell components.^10^ The preponderance of these biological factors within the micro‐environment, together with the expression of their receptors in cells of different lineages, are proof of mutual regulation between bone and nerves. Neurotransmitters, neuropeptides, neurotrophins and neuronal guidance factors, which are regulated by both skeletal tissues and peripheral nerves,^11^, ^12^ attach to receptors expressed by cells of the nervous system as well as bone lineage cells.^13^, ^14^ These signalling molecules function as mediators between the nervous system and the skeleton.

Dental pulp stem cells (DPSCs) are commonly utilized in neural regeneration and bone tissue engineering due to their easy isolation, lack of ethical controversy, low immunogenicity and reduced rates of transplantation rejection.^15^ Due to DPSCs' multidirectional differentiation potential and easy accessibility, their application in bone tissue engineering is currently under extensive exploration. DPSCs have demonstrated clinical potential not only in dentistry but also in the treatment of various diseases, including craniofacial bone defects, muscle regeneration, myocardial infarction, Alzheimer's disease, nervous system injuries, Parkinson's disease, stress urinary incontinence, osteoarthritis and liver diseases.^16^, ^17^, ^18^, ^19^, ^20^, ^21^, ^22^, ^23^, ^24^

This study aimed to investigate whether co‐administration of DPSCs and human umbilical vein endothelial cells (HUVECs) could enhance the therapeutic effects of stem cells for critical hindlimb ischemia, compared to the effects of either DPSCs or HUVECs alone. The combination therapy of DPSCs and HUVECs resulted in significantly higher blood flow and reduced ischemic damage compared to the individual administration of DPSCs or HUVECs. The enhanced therapeutic effects observed in the DPSCs+HUVECs group were associated with a significantly greater number of microvessels in the ischemic tissue compared to the other groups. in vivo proliferation and tube formation assays demonstrated that VEGF present in the conditioned media of DPSCs facilitated the proliferation and formation of vessel‐like tubes by HUVECs.^25^ Other research has shown that rat bone marrow‐derived MSCs and HUVECs were co‐cultured on poly (lactic‐co‐glycolic acid) (PLGA) inverse opal scaffolds to promote vascularization for bone tissue regeneration.^26^ The researchers demonstrated that the presence of HUVECs in three‐dimensional spheroid cultures of collagen/fibronectin gels induced the formation of a pro‐vascular network, increased cell viability and proliferation, enhanced the osteogenic differentiation of the spheroids and increased the deposition of bone minerals by incorporating the HUVECs within the mesenchymal stem cell spheroids. In summary, mesenchymal stem cell/human umbilical vein endothelial cell spheroid‐loaded hydrogels provided a highly suitable 3D microenvironment for bone tissue formation, which can be utilized in various applications.^27^ Wang et al. treated DPSCs with extracellular vesicles (EVs) derived from Schwann cells (SCs). The results showed a clear increase in the proliferation, migration and osteogenic differentiation of DPSCs. SC‐EVs also promoted neurite outgrowth and neuron migration of rat dorsal root ganglia, as well as vessel formation in vivo. In an in vivo model of subcutaneous, SC‐EVs enhanced the recruitment of endogenous vascular endothelioid‐like cells and MSCs and promoted the formation of a pulpo‐dentinal complex‐like structure.^28^ Omi et al. found that DPSCs‐conditioned media promoted the neurite outgrowth of dorsal root ganglion neurons and increased the viability and myelin‐related protein expression of SCs.^29^ Bone tissue is a highly vascularized and neurotized tissue where blood vessels and nerve fibres closely interact and enhance the development and function of each other, eventually supporting bone development and fracture healing. Therefore, we envisioned that co‐culture of DPSCs with HUVECs and SCs could promote bone repair and bone remodelling. Our previous experiments showed that co‐culture of DPSCs with HUVECs and SCs can enhance the ability of neurovascularization and bone regeneration in vivo. In addition, we used a transwell indirect co‐culture system, in which HUVECs and SCs were in the upper chamber and DPSCs were in the lower chamber, and we detected the upregulation of nerve growth factor through examination of protein expression and cytokine secretion in the culture medium.

Nerve growth factor (NGF) plays a key role in regulating the survival, growth and differentiation of nerve cells through its two receptors: transmembrane tyrosine kinase A (TrkA), which binds to NGF with high affinity, and p75 neurotrophic factor receptor (p75NTR), a member of the tumour necrosis factor receptor superfamily, which exhibits low affinity for NGF.^30^ Extensive research has explored the effects of NGF and its receptors, TrkA and p75NTR, on nerve cells.^31^ Increasing evidence indicates that NGF, TrkA and p75NTR are involved in diverse aspects of MSC functions, encompassing survival, growth, differentiation and angiogenesis. It is widely believed that the regulatory effects of NGF on MSCs are primarily achieved through its interaction with TrkA.^32^, ^33^ Additionally, as a pivotal factor in bone regeneration, NGF promotes osteoblast mitosis, contributing to bone formation and ossification, and is widely expressed in bone marrow MSCs, regional callus, osteoclasts, chondrocytes, synovial fibroblasts and other skeletal cells.^34^, ^35^, ^36^

In this study, we investigated the effect of exogenous NGF on the osteogenic differentiation of DPSCs, which is associated with the activation of MEK/ERK signalling pathways. Furthermore, we knocked down TrkA and confirmed the interaction between NGF and TrkA, and further revealed the positive influence of TrkA on the osteogenic differentiation of DPSCs.

## 2 MATERIALS AND METHODS

### 2.1 Isolation and Culture of DPSCs

This study was approved by the Research Ethics Committee of School of Stomatology, Shandong University. Extracted healthy first or second premolars and third molars from donors aged from 18 to 23 years were collected with their and their parents' informed consent. The associated information was shown in Table S1. All donors were healthy and had no systemic diseases. All collected teeth had intact roots without caries or periodontitis. The pulp was removed from the crown and the roots under sterile conditions and then cut into 1 mm^3^ pieces. Tissue fragments were digested in a solution of 3 mg/mL collagenase type I (Solarbio, Beijing, China) and 4 mg/mL dispase (Roche, Basel, Switzerland) for about 60 min and mixed well every 10 min. Single‐cell suspensions were obtained by passing the cells through a 70‐mm strainer (BD Labware, Franklin Lakes, NJ, USA), seeded into 25 cm^2^ culture flask, and maintained in the α‐modification of Eagle's medium (α‐MEM; HyClone, Logan, Utah, USA) containing 20% foetal bovine serum (FBS; Invitrogen, Carlsbad, CA, USA), 0.1 mg/mL penicillin–streptomycin (Biosharp, Hefei, China), which was termed as basal medium. Cells were cultured in an incubator at 37°C in 5% CO2. The culture medium was refreshed every 2–3 days. Passages 3–5 of DPSCs were used in the following studies.

### 2.2 Identification of DPSCs

The surface markers of DPSCs were analysed by flow cytometry (BD, Franklin Lakes, NJ, USA) according to the manufacturer's instructions. After washing with phosphate buffer solution (PBS) twice, the cells were incubated with monoclonal antibodies against human CD34 (Abcam, Cambridge, UK), CD45 (Abcam, Cambridge, UK), CD146 (Abcam, Cambridge, UK), STRO‐1 (R&D Systems, Minneapolis, USA), CD73 (Abcam, Cambridge, UK) and CD90 (Abcam, Cambridge, UK) at 4°C for 30 min and analysed by flow cytometry.

To test their potent multilineage differentiation, DPSCs were cultured in the osteogenic‐inducing medium, i.e. basal medium supplemented with 50 mg/L vitamin C (Wako Chemical, Tokyo, Japan), 10 mmol/L sodium β‐glycerophosphate (Sigma‐Aldrich, St. Louis, MO, USA) and 10 nmol/L dexamethasone (Sigma‐Aldrich, St. Louis, MO, USA), or in the adipogenic‐inducing medium, i.e. basal medium supplemented with 0.5 mmol/L isobutyl‐methylxanthine (Sigma‐Aldrich, St. Louis, MO, USA), 60 μmol/L indometacin (Sigma‐Aldrich, St. Louis, MO, USA), 0.5 μmol/L hydrocortisone(Sigma‐Aldrich, St. Louis, MO, USA) and 10 μg/mL insulin (Sigma‐Aldrich, St. Louis, MO, USA) or in the chondrogenic‐inducing medium(OriCell® Cyagen Biosciences, Guangzhou, China), i.e. basal medium supplemented with cells chondrogenic differentiation I, cells chondrogenic differentiation II. After 21 days of osteogenic/adipogenic differentiation, the cells were washed 3 times with PBS, fixed with 4% paraformaldehyde solution for 15 min, and then were stained with Alizarin Red S staining kit (Solarbio, Beijing, China) and Oil Red O staining kit (Solarbio, Beijing, China), respectively. The Alcian blue staining was performed post‐sectioning after 21 days of chondrogenic differentiation.

### 2.3 Co‐culture of DPSCs, HUVECs and SCs

The six‐well plate transwell permeable supports (Corning, Tewksbury, MA, USA) with a 0.4 μm polycarbonate membrane were used in the co‐culture system to separate DPSCs, HUVECs and SCs into the different compartments. HUVECs (ScienCell, Calsbad, CA, USA) were cultured in endothelial cell medium (ScienCell, Calsbad, CA, USA) supplemented with 10% FBS and 0.1 mg/mL penicillin–streptomycin in a humidified atmosphere containing 5% CO_2_ at 37°C. SCs (ScienCell, Calsbad, CA, USA) were cultured in Schwann cell medium (ScienCell, Calsbad, CA, USA) supplemented with 10% FBS and 0.1 mg/mL penicillin–streptomycin in a humidified atmosphere containing 5% CO_2_ at 37°C. HUVECs (1.0 × 10^4^/well) /SCs (1.0 × 10^4^/well)/ HUVECs (1.0 × 10^4^/well) + SCs (1.0 × 10^4^/well) were inoculated into the upper chamber. DPSCs (1.0 × 10^5^/well) were inoculated in the lower chamber. The co‐culture experiment was divided into the following four groups: (1) DPSCs alone; (2) DPSCs+HUVECs (10:1); (3) DPSCs+SCs (10:1); (4) DPSCs+HUVECs+SCs (10:1:1). After 3 days, the DPSCs in the lower chambers were collected for further experiments.

### 2.4 Proliferation assay

Dental pulp stem cells (5.0 × 10^3^ cells/well) were seeded into 96‐well plates for 24 h and treated with 50 ng/mL NGF (11050‐HNAC; Sino Biological, Beijing, China) for 1–7 days. The proliferation profiles of DPSCs were measured by using Cell Counting Kit‐8 (CCK‐8; Dojindo Laboratories, Japan) following the instruction of manufacturer. Briefly, the culture medium was removed and changed into 100 μL α‐MEM mixed with 10 μL CCK8, and the plate was incubated for 1 h at 37°C. Next, a multiwell spectrophotometer (Molecules Devices, Sunnyvale, CA, USA) was used to detect the optical density (OD) value at 450 nm.

### 2.5 Osteogenic differentiation

Dental pulp stem cells were seeded at a density of 2.0 × 10^5^cells/well in 6‐well culture plates and incubated in osteogenic differentiation medium as mentioned above with either 50 ng/mL of NGF and/or 10 μM of Ro 08–2750 (HY‐108466; MedChemExpress, Shanghai, China) and/or 10 μM U0126 (HY‐12031; MedChemExpress, Shanghai, China) at 37°C for 7 days. The medium was changed twice per week. Osteogenic differentiation was detected by ALP staining (Solarbio, Beijing, China) and the assessment of ALP activity (Nanjing Jiancheng Bio‐engineering Institute; Beyotime, Nanjing, China) according to the manufacturer's instructions, and the absorbance was measured by microplate reader at a wavelength of 405 nm.

### 2.6 Small interfering RNA transfection

Small interfering RNA (siRNA) for TrkA (HH20220531GX‐SI05) and negative control siRNA were purchased from Hanbio RNA Technologies (Hanbio, Shanghai, China). DPSCs (0.2 × 10^6^ cells/well) seeded in six‐well plates were transfected with 50 nM siRNA with the use of Lipofectamine2000 Reagent (Invitrogen, Carlsbad, CA, USA) according to the manufacturer's instructions. After interference for 6 h, osteogenic differentiation fluid was added for 72 h. Target gene downregulation was confirmed by quantitative reverse transcription polymerase chain reaction (qRT‐PCR) and Western blot, respectively.

### 2.7 RNA extraction and qRT‐PCR

Total RNA was isolated from DPSCs using RNAiso TM Plus (Accurate Biology, Hunan, China) according to the manufacturer's protocol. Extracted total RNA was reverse‐transcripted using the Prime Script RT Reagent Kit with gDNA Eraser (Accurate Biology, Hunan, China). The relative level of RNA was detected using the LightCycler‐480 system (Roche Diagnostics GmbH, Mannheim, Germany) and TB Green Premix Ex Taq II (Accurate Biology, Hunan, China). GAPDH was used as internal controls. The PCR reaction conditions were as follows: 95°C for 30 s, then 55 cycles of 95°C for 10 s and 60°C for 30 s. The $2^{-∆∆C_{q}}$ method was used for comparative quantitation. Sequences of primers are shown in Table 1. All PCR reactions were performed in triplicate.

**TABLE 1:** Primer sequences of osteogenic genes and TrkA.

Primer name	Sense primers (5′–3′)	Antisense primers (3′–5′)
ALP	TCCATCTGTAAAGGGCGGTAAT	AATACCAGCTACGCTGCATCAAG
RUNX2	GTTTCACCTTGACCATAACCGT	GGGACACCTACTCTCATACTGG
COL1	GCTGATGATGCCAATGTGGTT	CCAGTCAGAGTGGCACATCTTG
OPN	ATCTGGACTGCTTGTGGCTG	GCCGTGGGAAGGACAGTTAT
NGF	TCTGTGGCGGTGGTCTTATC	GCAAGCGGTCATCATCCCAT
TrkA	ATCCCTGTCTCCTTCTCGCC	CACCGAGACCCCAAAAGGTG
GAPDH	TCATGGGTGTGAACCATGAGAA	GGCATGGACTGTGGTCATGAG

Abbreviations: ALP, alkaline phosphatase; COL1, collagen I; GAPDH, glyceraldehyde 3‐phosphate dehydrogenase; NGF, nerve growth factor; OPN, Osteopontin; RUNX2, runt‐related transcription factor 2; TrkA, tyrosine kinase receptor A.

### 2.8 Western blot analysis

Dental pulp stem cells were collected and lysed in RIPA reagent (Solarbio, Beijing, China) containing 1% PMSF (Solarbio, Beijing, China). Protein samples were separated in a 10% sodium dodecyl sulfate‐polyacrylamide gel electrophoresis and transferred to a 0.2 μm polyvinylidene fluoride membrane (Millipore, Burlington, MA, USA). After blocking in 5% skimmed milk, membranes were incubated overnight at 4°C with the following primary antibodies: rabbit anti‐RUNX2 (1:1000, Lot # 8486S; Cell Signalling Technology, MA, USA), rabbit anti‐ALP (1:1000, 11,187‐1‐AP; Proteintech, Rosemont, IL, USA), rabbit anti‐COL1 (1:1000, 14,695‐1‐AP; Proteintech, Rosemont, IL, USA), rabbit anti‐OPN (1:1000, 22,952‐1‐AP; Proteintech, Rosemont, IL, USA), rabbit anti‐phospho‐MEK (p‐MEK, 1:1000, bsm‐52176R; Bioss, Beijing, China), rabbit anti‐MEK (t‐MEK, 1:500, WL03328; Wanleibio, Shenyang, China), rabbit anti‐phospho‐ERK (p‐ERK, 1:300, WLP1512; Wanleibio, Shenyang, China), rabbit anti‐ERK (t‐ERK, 1:500, WLO1864; Wanleibio, Shenyang, China), rabbit anti‐TrkA (1:500, WLO1390; Wanleibio, Shenyang, China) and rabbit anti‐GAPDH (1:1000, 10,494‐1‐AP; Proteintech, Rosemont, IL, USA). After washing in TBS (Solarbio, Beijing, China) with Tween‐20 (Solarbio, Beijing, China), membranes were incubated at 25°C for 1 h with peroxidase‐conjugated anti‐rabbit IgG (1:10,000, SA00001‐2; Proteintech, Rosemont, IL, USA). An ECL chromogenic substrate (Millipore, Burlington, MA, USA) was used for the detection of immunoreactive bands. The Image J software (NIH, Bethesda, MD, USA) was used to quantify the densitometric data. Analysis of immunoreactive bands used GAPDH as the control.

### 2.9 Statistical analysis

Data are presented as mean ± SD of triplicates of at least three independent experiments. The experimental data were statistically analysed by normality test. Differences were analysed by one‐way ANOVA or a two‐tailed unpaired Student's *t*‐test. Statistical significance was analysed using GraphPad Prism software 8.0 (GraphPad Software, La Jolla, CA, USA). A *p* value < 0.05 indicated that the difference was statistically significant.

## 3 RESULTS

### 3.1 Cell culture of DPSCs

**FIGURE 1:**
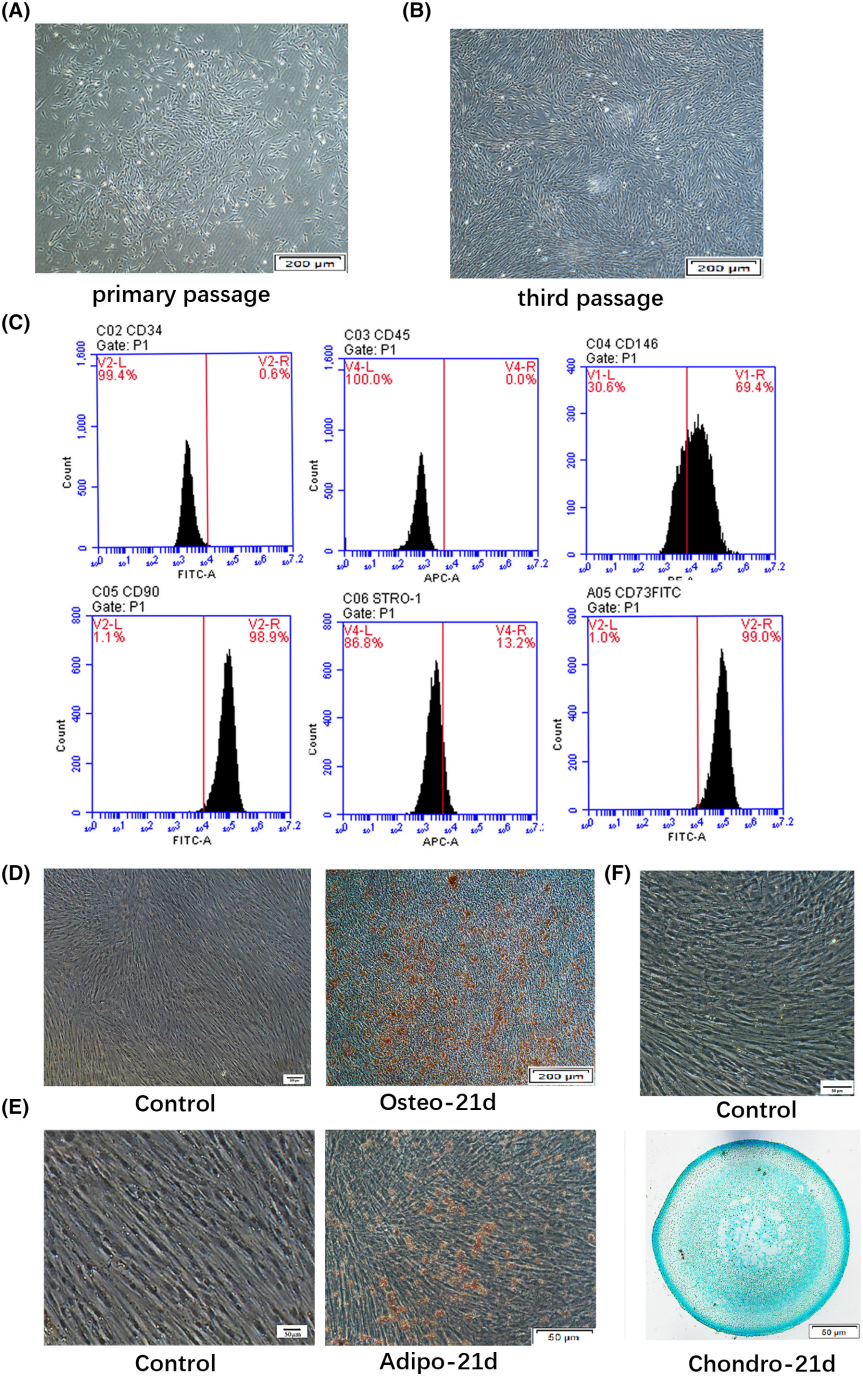
Dental pulp stem cells culture and identification. Cell morphology of DPSCs at primary (A) and third passage (B). Scale bar = 200 μm. (C) Flow cytometry analysis showed that DPSCs were negative for CD34 and CD45, whereas positive for CD146, CD90, CD73 and STRO‐1. (D) The Alizarin Red S staining showed the increased level of calcium nodules after osteogenic induction for 21 days. Scale bar = 200 μm. (E) DPSCs were found to possess the potential to develop into Oil red O‐positive lipid‐laden fat cells following 21 days of culture with an adipogenic‐inductive medium. Scale bar = 50 μm. (F) DPSCs were found positive for Alcian blue staining following 21 days of culture with a chondrogenic inductive medium. Scale bar = 50 μm.

All cultured DPSCs had the typical spindle‐shaped structure (Figure 1A,B). DPSCs were negative for CD34 (0.6%) and CD45 (0.0%), which are cell surface markers of haematopoietic lineage cells, whereas positive for CD146 (69.4%), CD90 (98.9%), CD73 (99.0%) and STRO‐1 (13.2%), three typical markers of culture‐expanded MSCs (Figure 1C). In addition, Alizarin Red S staining (Figure 1D), Oil Red O staining (Figure 1E) and Alcian blue staining (Figure 1F) showed the increased level of calcium nodules, lipid‐laden fat cells and Alcian blue positive areas from DPSCs after osteogenic, adipogenic and chondrogenic induction, respectively, implying the multi‐potent differentiation capabilities of DPSCs.

### 3.2 NGF promoted proliferation of DPSCs

**FIGURE 2:**
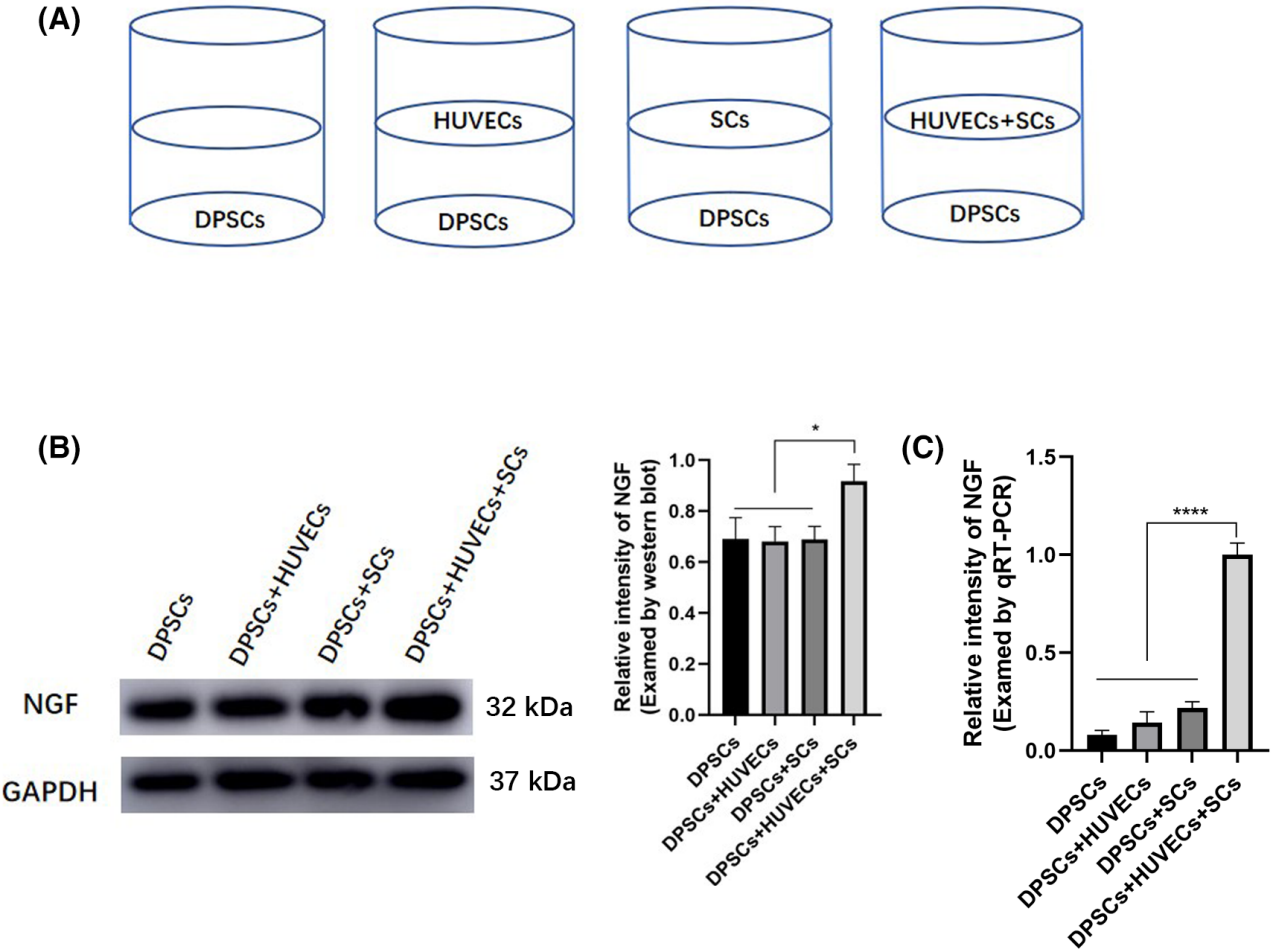
Nerve growth factor promoted proliferation of DPSCs. (A) The co‐culture pattern of DPSCs, HUVECs and SCs in transwell system at indicated ratio. NGF was up‐regulated in the co‐culture of DPSCs+HUVECs+SCs confirmed by western blot analysis, *n* = 3 (B) and qRT‐PCR analysis (C). *p < 0.05, **p < 0.01, ***p < 0.001, ****p < 0.0001.

In our preliminary experiments, DPSCs were co‐cultured with different quantities of HUVECs and SCs (DPSCs:HUVECs: SCs = 1:1:1, 2:1:1, 5:1:1, 10:1:1, respectively) under osteogenic induction. The results showed that the expression of RUNX2 were significantly increased in the 10:1:1 group compared with other groups (data not shown). Thus, the cell ratio of DPSCs:HUVECs:SCs at 10:1:1 was used in the following experiments. DPSCs, HUVECs and SCs were co‐cultured in transwell culture system at indicated ratios as shown in the diagram (Figure 2A). Western blot analysis confirmed the upregulated protein level of NGF in the co‐culture of DPSCs+HUVECs+SCs group (Figure 2B). The expression level of NGF showed a 1.3‐fold increase compared to the other three groups. qRT‐PCR analysis confirmed the upregulated gene level of NGF in the co‐culture of DPSCs+HUVECs+SCs group (Figure 2C). The expression level of NGF showed a 1.89‐fold increase compared to the other three groups. To determine the effect of NGF on DPSCs proliferation, we treated DPSCs with NGF (50 ng/mL) for 1–7 days and found that the proliferation activity of DPSCs in the presence of 50 ng/mL NGF increased gradually in a time‐dependent manner by using CCK‐8 test (Figure S1).

### 3.3 NGF promoted osteogenic differentiation of DPSCs

**FIGURE 3:**
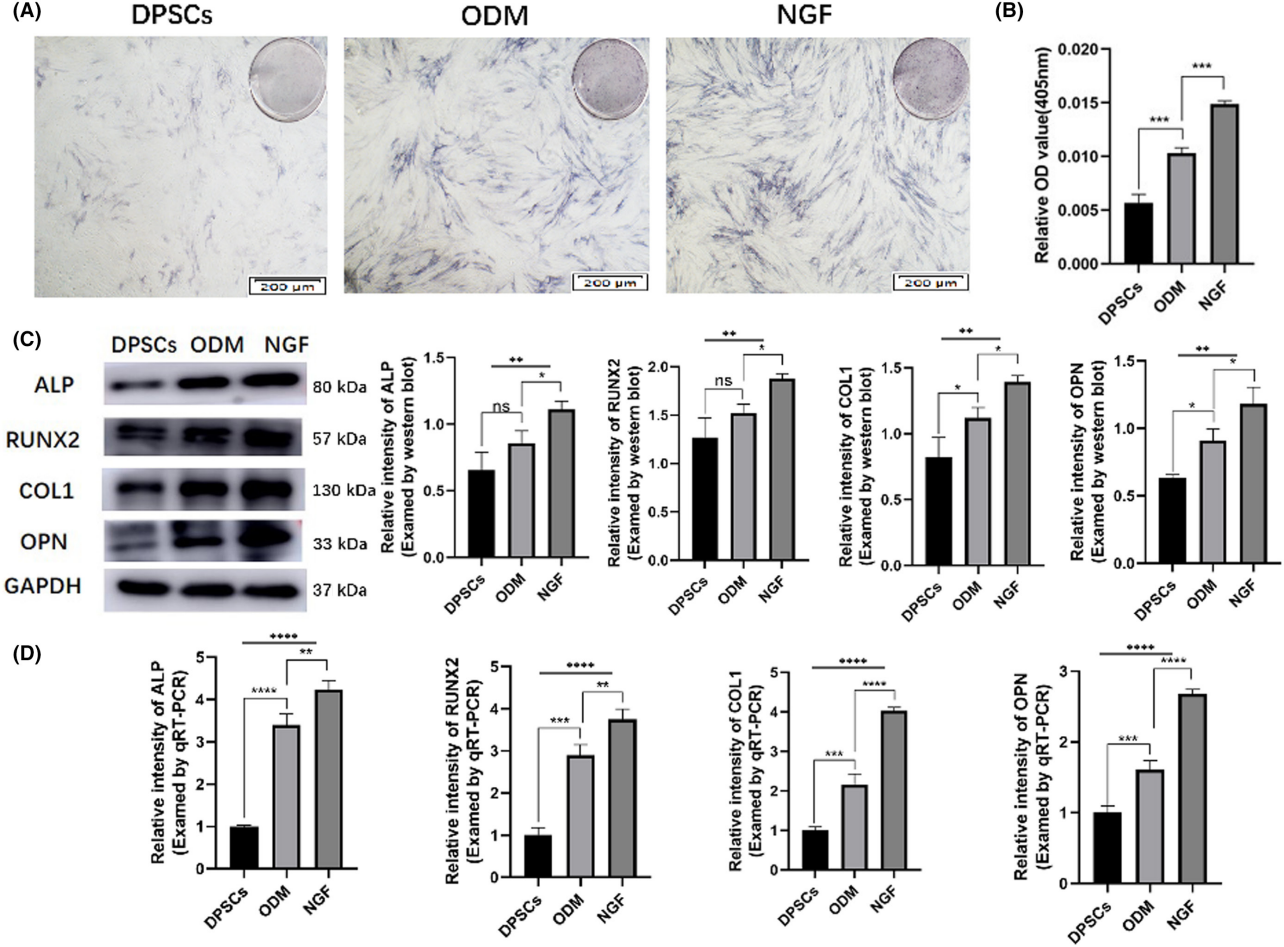
NGF promoted osteogenic differentiation of DPSCs. NGF at 50 ng/mL was capable of enhancing the ALP staining (A), ALP activity (B) of the DPSCs and increasing the protein (C) and gene (D) expression levels of ALP, RUNX2, COL1 and OPN. *p < 0.05, **p < 0.01, ***p < 0.001, ****p < 0.0001. ns, no significance. ODM, osteogenic differentiation medium.

Dental pulp stem cells were seeded onto 6‐well cell culture plates with or without NGF (50 ng/mL) at a density of 2.0 × 10^5^ cells per well for 7 days in osteogenic induction medium. We first performed the ALP staining and tested the activity of ALP, which is an essential enzyme for bone formation and an early marker of osteoblast differentiation and functional maturation, and the results showed that treatment with 50 ng/mL NGF promoted dyeing deepening of ALP (Figure 3A) and dramatically enhanced the ALP activity (Figure 3B) compared with the control groups. After NGF treatment, the ALP activity showed a 1.44‐fold increase compared to the ODM group.

To further investigate the effects of NGF on osteogenic differentiation in DPSCs, the protein and gene expression levels of ALP, RUNX2, COL1 and OPN were detected by Western blot and qRT‐PCR, respectively. The data showed that NGF treatment significantly increased the protein (Figure 3C) and gene (Figure 3D) expression levels of the above‐mentioned markers. Taken together, these results demonstrated that NGF significantly promoted osteogenic differentiation of DPSCs under osteogenic induction.

### 3.4 MEK/ERK signalling pathway was involved in the NGF‐mediated promotion of DPSCs osteogenic differentiation

**FIGURE 4:**
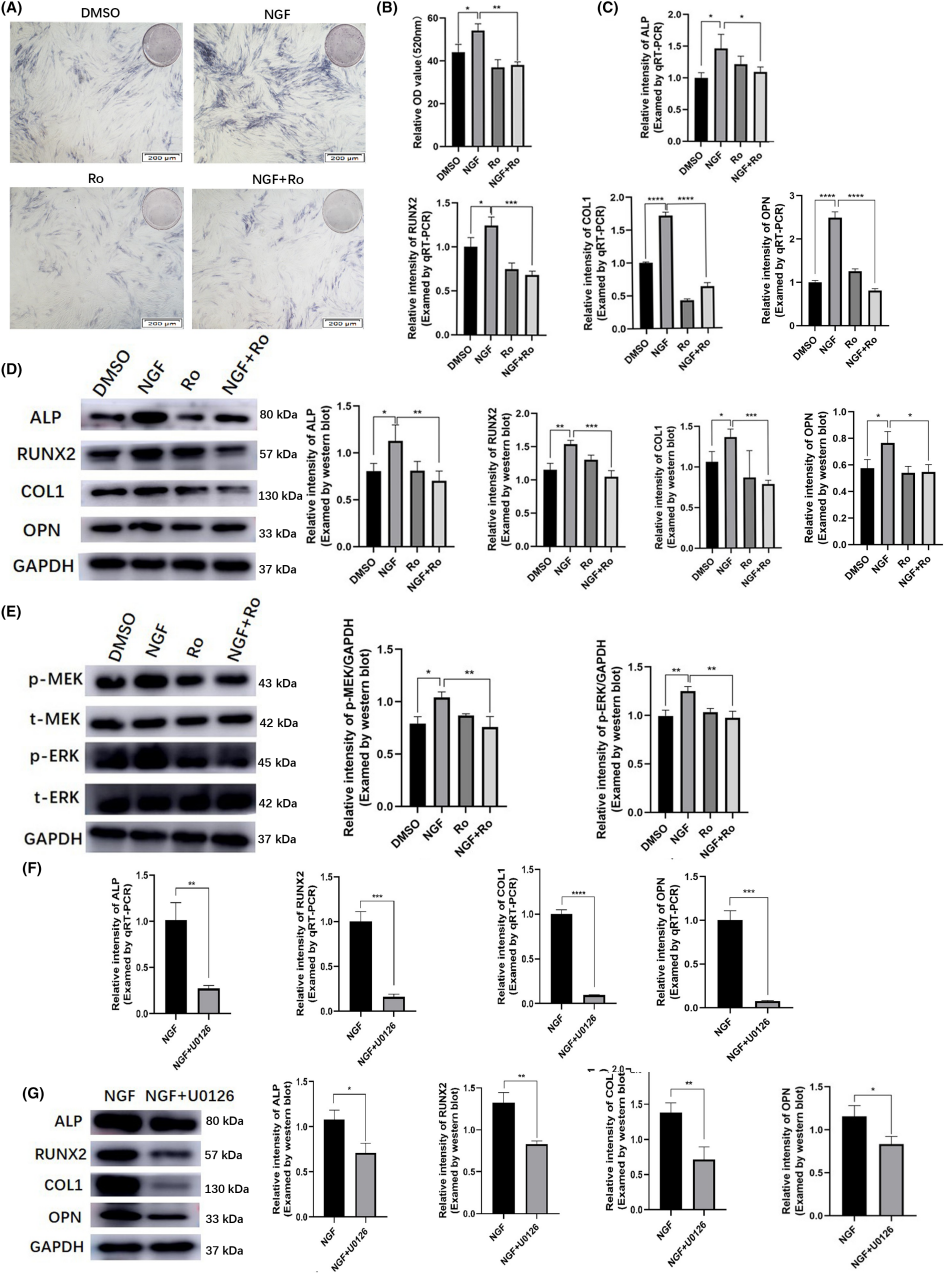
MEK/ERK signalling pathway was involved in the NGF‐mediated promotion of DPSCs osteogenic differentiation. Treatment of DPSCs with Ro 08‐2750 (10 μM), a NGF inhibitor, could decrease the ALP staining (A), ALP activity (B) of the DPSCs and reducing the gene (C) and protein (D) expression levels of ALP, RUNX2, COL1 and OPN. (E) Western blot analysis demonstrated the levels of *p*‐MEK and *p*‐ERK were increased after NGF treatment, whereas the presence of Ro 08‐2750 decreased the *p*‐MEK and *p*‐ERK levels. U0126, the MEK/ERK pathway inhibitor, could significantly inhibited NGF‐mediated DPSCs' osteogenic differentiation manifested by the downregulation of ALP, RUNX2, COL1 and OPN through qRT‐PCR (F) and Western blot (G) analysis, *n* = 3. Scale bar = 200 μm. *p < 0.05, **p < 0.01, ***p < 0.001, ****p < 0.0001. Ro, Ro 08‐2750. DMSO group, served as control group, in which DMSO is the solvent of Ro 08‐2750.

After the confirmation of NGF‐mediated osteogenic differentiation of DPSCs, we treated DPSCs with Ro 08–2750 (10 μM), a NGF inhibitor that can block the binding of NGF to its receptor on the cells. The data demonstrated that Ro 08–2750 treatment decreased the ALP staining (Figure 4A, Figure S2) and ALP activity (Figure 4B) in NGF + Ro 08–2750 group. After Ro treatment, the ALP activity showed a 1.43‐fold increase compared to the NGF + Ro 08–2750 group (NGF vs. NGF + Ro: *p* = 0.001). Not surprisingly, compared with the NGF treatment, presence of 10 μM Ro 08–2750 dramatically down‐regulated the mRNA (Figure 4C) and protein (Figure 4D) expression profiles of ALP, RUNX2, COL1 and OPN. We further analysed the underlying mechanism whereby NGF increased osteogenic differentiation of DPSCs. Western blot analysis revealed that after NGF treatment, the levels of *p*‐MEK and *p*‐ERK expression were significantly increased. Meanwhile, the expression levels of total MEK were not changed. Moreover, inhibition of NGF by addition of Ro 08–2750 decreased the levels of *p*‐MEK and *p*‐ERK (Figure 4E). After U0126 treatment, compared with the NGF treatment, presence of 10 μM U0126 dramatically down‐regulated the mRNA (Figure 4F) and protein (Figure 4G) expression profiles of ALP, RUNX2, COL1 and OPN, respectively.

**FIGURE 5:**
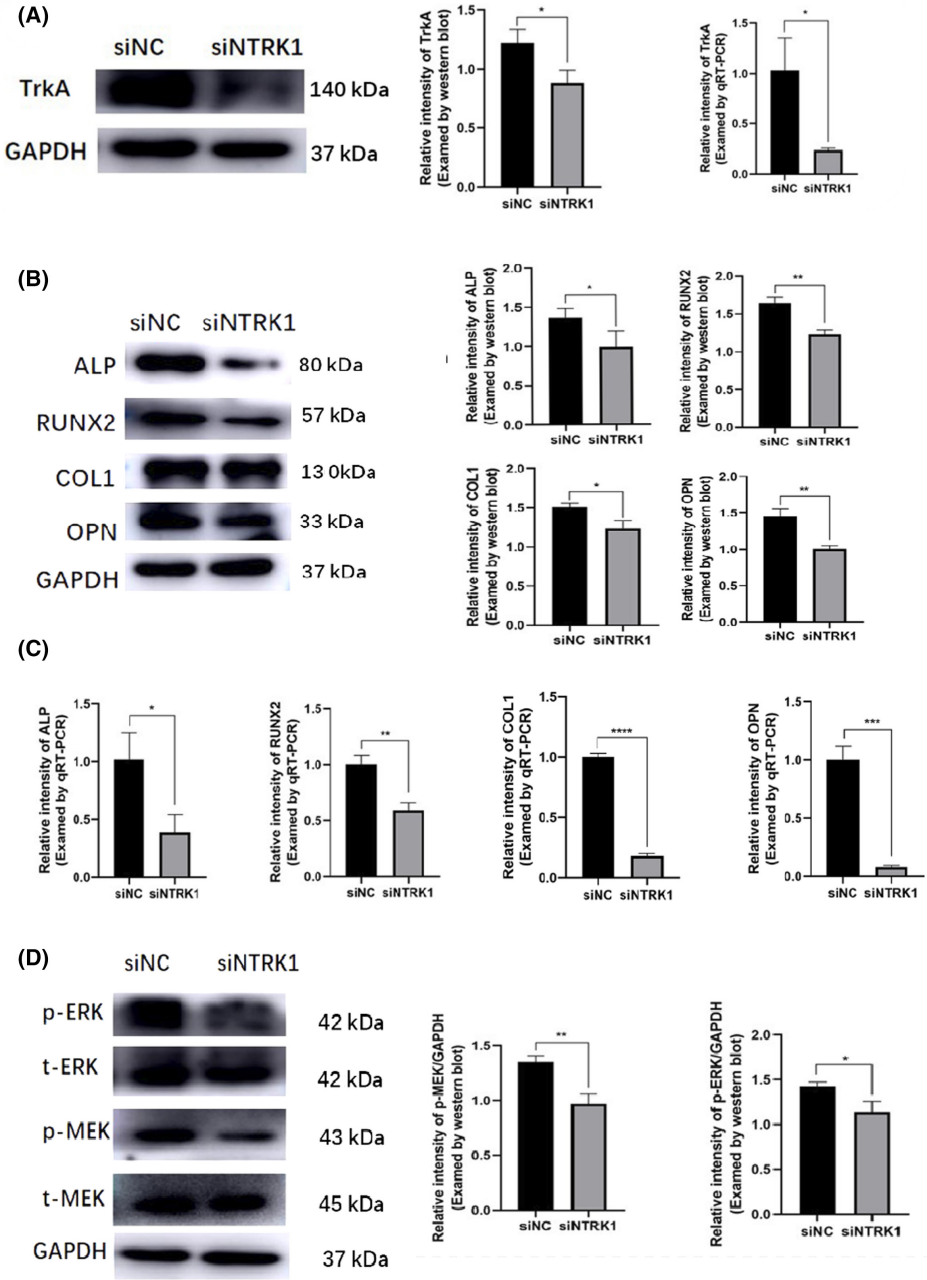
Knockdown of TrkA suppressed NGF‐induced osteogenic differentiation of DPSCs (A) Target gene downregulation was confirmed by qRT‐PCR and Western blot, *n* = 3. TrkA knockdown (siNTRK1) could significantly inhibited DPSCs' osteogenic differentiation manifested by the downregulation of ALP, RUNX2, COL1 and OPN through Western blot (B) and qRT‐PCR analysis (C), *n* = 3. (D) TrkA knockdown was able to inhibit the levels of *p*‐MEK and *p*‐ERK. *p < 0.05, **p < 0.01, ****p < 0.0001.

To further explore the key role of NGF in osteogenic differentiation of DPSCs, siRNAs were used in DPSCs to specifically knock down the expression of TrKA, the functional receptor for NGF and encoded by NTRK1 protein and gene (Figure 5A). TrkA knockdown (siNTRK1) could significantly inhibited DPSCs' osteogenic differentiation, manifested by the downregulation of ALP, RUNX2, COL1 and OPN through Western blot (Figure 5B) and qRT‐PCR analysis (Figure 5C). Additionally, TrkA knockdown was able to inhibit the levels of *p*‐MEK and *p*‐ERK (Figure 5D).

**FIGURE 6:**
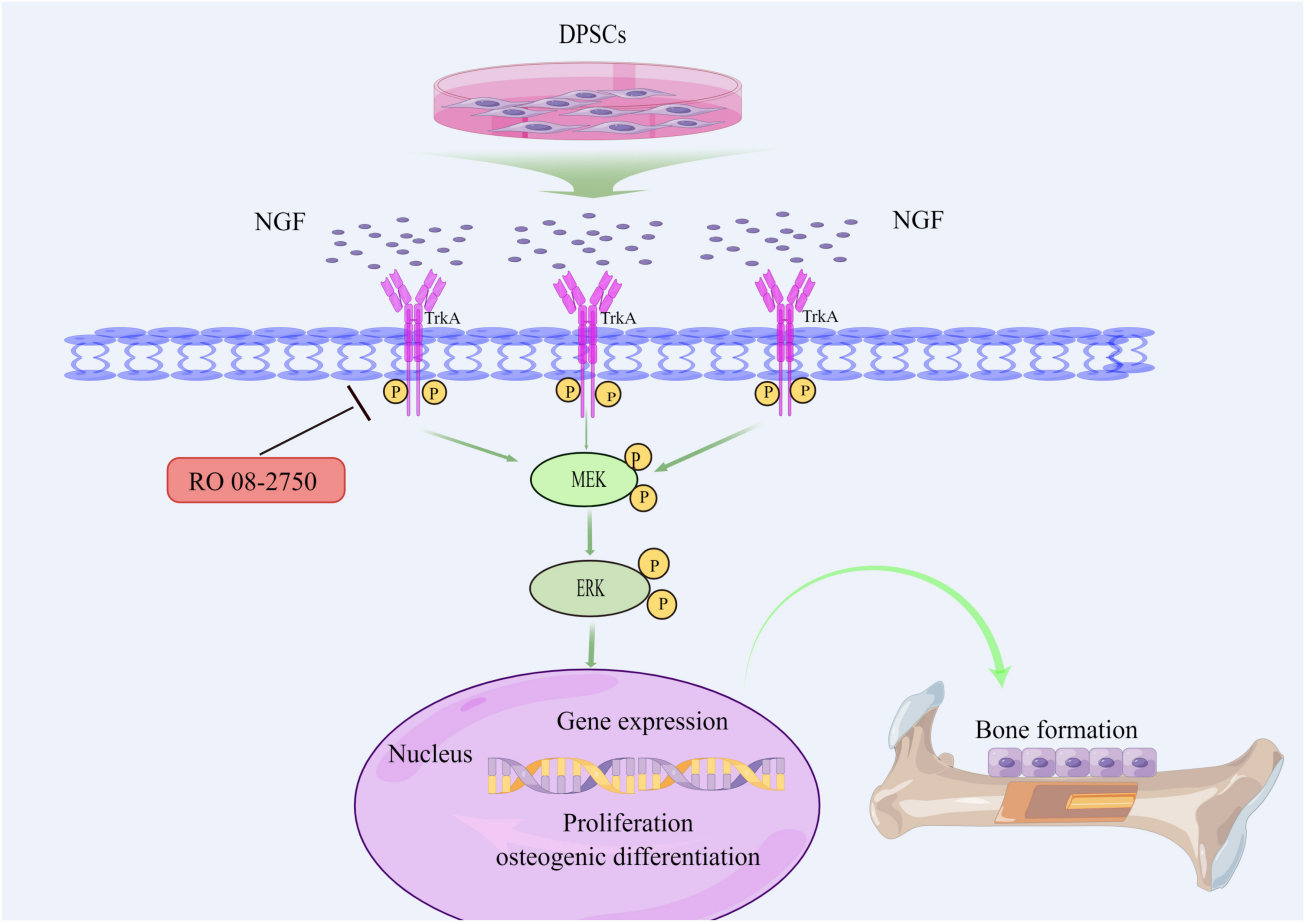
NGF‐mediated osteogenic differentiation of DPSCs through MEK/ERK signalling pathways.

All these results proved that NGF could promote the osteogenic differentiation of DPSCs via the MEK/ERK signalling pathway (Figure 6).

## 4 DISCUSSION

Similar to bone marrow MSCs, DPSCs have the ability to form clones, express MSC markers and differentiate into cells with osteoblastic, chondrocytic and adipocytic characteristics under specific culture conditions. This makes them a promising source of MSCs for cell therapy and regenerative medicine.^37^, ^38^ The commitment and differentiation of DPSCs into specific mature cell types, such as osteocytes, is a complex process influenced by chemical stimuli, growth factors, cytokines and extracellular matrix components.^39^ Because of their excellent biological functions, accessibility and minimally invasive harvesting process, DPSCs have been utilized in several clinical studies, indicating their significant potential in tissue repair and regeneration.^40^ DPSCs have demonstrated robust angiogenic and osteogenic potential in both in vivo and in vivo settings, being capable of direct differentiation into or interaction with endothelial cells and osteoblasts.^41^, ^42^ Although numerous studies have demonstrated the potential of DPSCs to enhance bone regeneration in vivo, the mechanism by which DPSCs promote regeneration and the possibility of large‐volume bone formation through DPSC‐based therapy are still under investigation.^43^ Therefore, it is crucial to investigate reliable and effective methods for promoting the osteogenic differentiation of DPSCs.

Bone tissue is highly vascularized and innervated.^4^ Clinically, bones lacking innervation or with weakened innervation are prone to fractures or bone‐related diseases, such as osteoporosis. NGF is a well‐studied neurotrophic factor and one of the first growth factors to be identified and characterised for its ability to stimulate the survival and growth of nerve cells.^44^, ^45^ Besides its crucial roles in the development and maintenance of the nervous system, NGF and its receptors are expressed in various cells and tissues. Thus, the functions of NGF are not limited to the nervous system. An increasing number of studies have demonstrated the important role of NGF in regulating the biological effects of MSCs.^46^ For example, NGF protected bone marrow MSCs against apoptosis, and transplanted NGF‐overexpressing MSCs exhibited increased cell migration in the ischemic brains of stroke rats, primarily mediated by the high‐affinity receptor TrkA.^47^ According to Luo et al., NGF significantly enhanced the proliferation of human umbilical cord mesenchymal stem cells (hUCMSCs) and mitigated cytotoxicity and apoptosis under hypoxic conditions. Furthermore, NGF promoted the paracrine effects of hUCMSCs on angiogenesis and cardiomyocyte protection.^48^ Transplantation of MSCs may effectively treat periodontitis, but high glucose limits their therapeutic effect in diabetes. NGF has functions of cell protection, anti‐apoptosis and immune regulation and may have potential applications in diabetic periodontitis.^49^ These studies affirmed NGF's ability to regulate the behaviour of MSCs.

The present study investigated whether NGF is involved in osteogenic differentiation of DPSCs. We set up different concentration gradients of NGF (25 ng/mL, 50 ng/mL and 100 ng/mL) by referring to relevant literature. Western blot results showed that the expression of ALP and COL1 osteogenic proteins was significantly increased in 50 ng/mL NGF group compared with other groups. So, we chose 50 ng/mL for the follow‐up experiment. The results of ALP staining and activity identification showed that DPSCs treated with 50 ng/mL had deeper ALP staining and higher ALP activity than those in the osteogenic induction group. Serving as indicators of osteogenic differentiation, ALP, RUNX2, COL1 and OPN manifested by their protein and mRNA expression profiles, also reflected the ability of NGF to induce osteogenic differentiation of DPSCs. The results showed that 50 ng/mL NGF could promote the differentiation of DPSCs into osteoblasts. It is well known that NGF achieves its biological functions by triggering downstream signals for targeting receptors.^50^ Herein, functional experiments demonstrated that cell osteogenic differentiation of DPSCs was markedly inhibited by TrkA knockdown. Therefore, we concluded that NGF promoted cell osteogenic differentiation of DPSCs through positively regulating TrkA expression. It cannot be ignored that DPSCs are extremely heterogeneous cell sources, and their biological responses largely depend on cell sub‐populations. Differences in cellular sub‐populations of DPSCs and different culture conditions, perhaps influencing the functional collaboration between the two receptors of NGF in DPSCs (TrkA and p75NTR), are worthy of further investigation.

We investigated the molecular mechanisms underlying osteogenic differentiation of DPSCs mediated by NGF. External growth factors regulate the complex process of cell proliferation and apoptosis, in which the MAPK pathways play a critical role and are involved in protein kinase cascades. MEK/ERK pathway is one of the most extensively characterised MAPK signalling pathways, reported to mediate osteogenic differentiation and induce osteogenesis.^42^, ^51^ As a MAPK kinase, MEK can phosphorylate ERK, ultimately enhancing its enzymatic activity. ERK has been shown to be a classical pathway associated with cellular proliferation, differentiation and survival. Subsequently, the activated ERK translocates to the nucleus and transactivates transcription factors, altering gene expression to promote cell population growth, differentiation, or mitosis.^52^ For instance, epiregulin could enhance cell proliferation in dental tissue‐derived MSCs by activating the MEK/ERK signalling pathways.^53^ Moreover, the MEK/ERK pathway influences the osteogenesis via modulating the phosphorylation and transcriptional expression of RUNX2.^54^ Previous studies demonstrated that NGF promotes the expression of fibroblast growth factor 2 in vivo via ERK/MAPK signalling pathways in human chondrocytes, further increasing angiogenesis mediated by TrkA.^55^ NGF modulates synaptic plasticity through the MAPK/ERK pathway, inducing the phosphorylation of synapsin I and regulating the interaction of synaptic vesicles with the cytoskeleton, promoting neurotransmitter release. As the treatment of bone defects presents an orthopaedic dilemma, our findings suggest that developing or selecting small‐molecule drugs or gene therapy to regulate DPSC‐mediated bone regeneration via MEK/ERK signalling may be a reliable approach, thereby facilitating bone tissue engineering.

Nerve growth factor is an important neural factor in bone biology, predominantly secreted from the densely distributed sensory neuronal fibres in bone and is essential for maintaining bone homeostasis and beneficial for clinical applications. Our study provided evidence for the role of the NGF/TrkA in the regulation of the osteogenic differentiation of DPSCs through MEK/ERK pathway, offering novel insights for the role of NGF in regulating cell differentiation and laying foundation for future therapeutic strategies on DPSCs‐regulated bone regeneration. However, the present study only conducted the in vivo experiments. Therefore, these findings require to be comprehensively confirmed in vivo, and animal models should be used to elucidate the NGF‐based DPSCs' regenerative potential in vivo before clinical application. Moreover, the precise regulation of DPSCs' osteogenic differentiation and bone regeneration and the underlying molecular mechanisms need to be further studied and discussed, which are the future’ research directions and goals.

## 5 CONCLUSIONS

Collectively, NGF was capable of promoting osteogenic differentiation of DPSCs through MEK/ERK signalling pathways, which may enhance the DPSCs‐mediated bone tissue regeneration.

## AUTHOR CONTRIBUTIONS

**Chen Cheng:** Conceptualization (equal); data curation (equal); formal analysis (equal); investigation (equal); methodology (equal); validation (equal); writing – original draft (lead); writing – review and editing (lead). **Shuai Tang:** Data curation (equal); formal analysis (equal); investigation (equal); methodology (equal); validation (equal); visualization (equal); writing – original draft (equal); writing – review and editing (equal). **Shuyue Cui:** Investigation (equal); methodology (equal); validation (equal). **Tong Yang:** Formal analysis (equal); investigation (equal); methodology (equal); validation (equal). **Lan Li:** Methodology (equal); validation (equal); visualization (equal). **Mingrui Zhai:** Formal analysis (equal); validation (equal); visualization (equal). **Fulan Wei:** Funding acquisition (equal); supervision (equal); writing – review and editing (supporting). **Gang Ding:** Conceptualization (equal); supervision (equal); writing – review and editing (supporting).

## CONFLICT OF INTEREST STATEMENT

No potential conflicts of interest were disclosed.

## Supporting information


Figure S1.

Figure S2.

Table S1.

## Data Availability

All data generated or analysed during this study are available from the corresponding authors on reasonable request.
