# Supplementary material for: Nerve growth factor promote osteogenic differentiation of dental pulp stem cells through MEK/ERK signalling pathways
Source: J Cell Mol Med. 2024 Feb 9;28(4):e18143. doi: 10.1111/jcmm.18143 (PMC10853700; doi:10.1111/jcmm.18143)
Supplement: Supplementary file 1 — Figure S1. [file JCMM-28-e18143-s002.docx]

**Supplementary Figure 1**

**
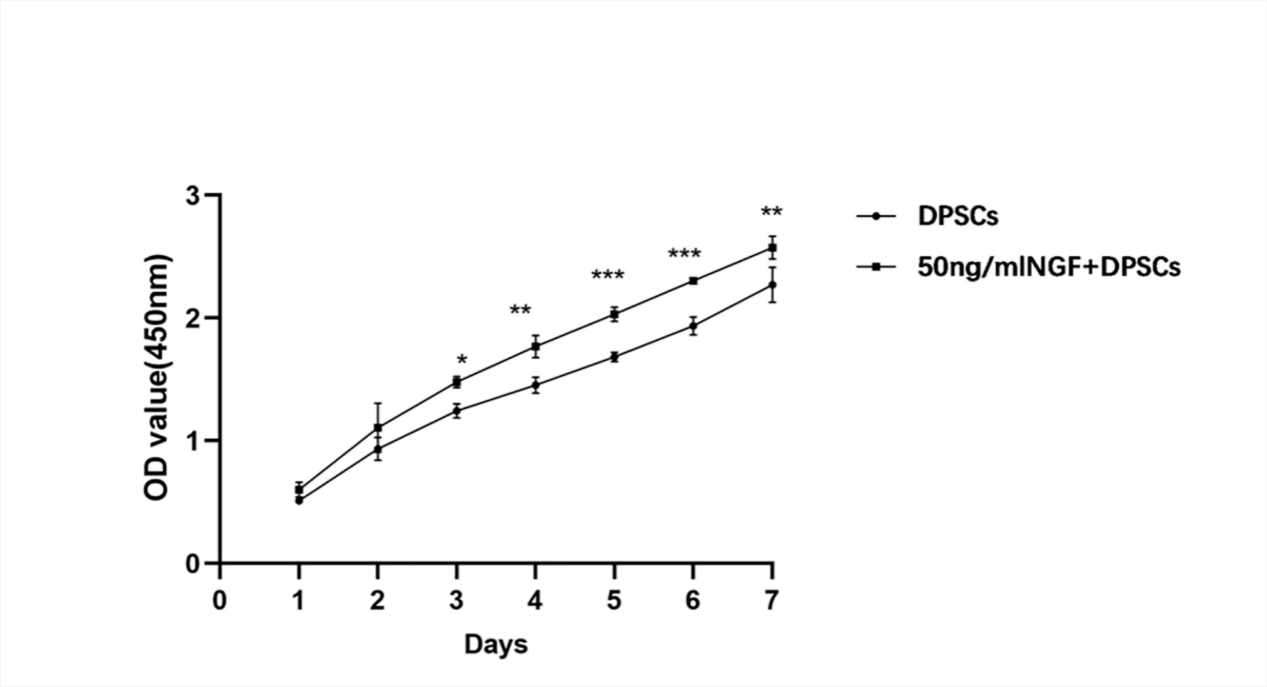
**

**Supplementary Figure 1** The CCK-8 examination showed that NGF (50ng/mL) increased the proliferation of DPSCs in a time-dependent manner. *p < 0.05, **p < 0.01, ***p < 0.001, ****p <0.0001.
