# Supplementary material for: Nerve growth factor promote osteogenic differentiation of dental pulp stem cells through MEK/ERK signalling pathways
Source: J Cell Mol Med. 2024 Feb 9;28(4):e18143. doi: 10.1111/jcmm.18143 (PMC10853700; doi:10.1111/jcmm.18143)
Supplement: Supplementary file 2 — Figure S2. [file JCMM-28-e18143-s001.docx]

**Supplementary Figure 2**

**
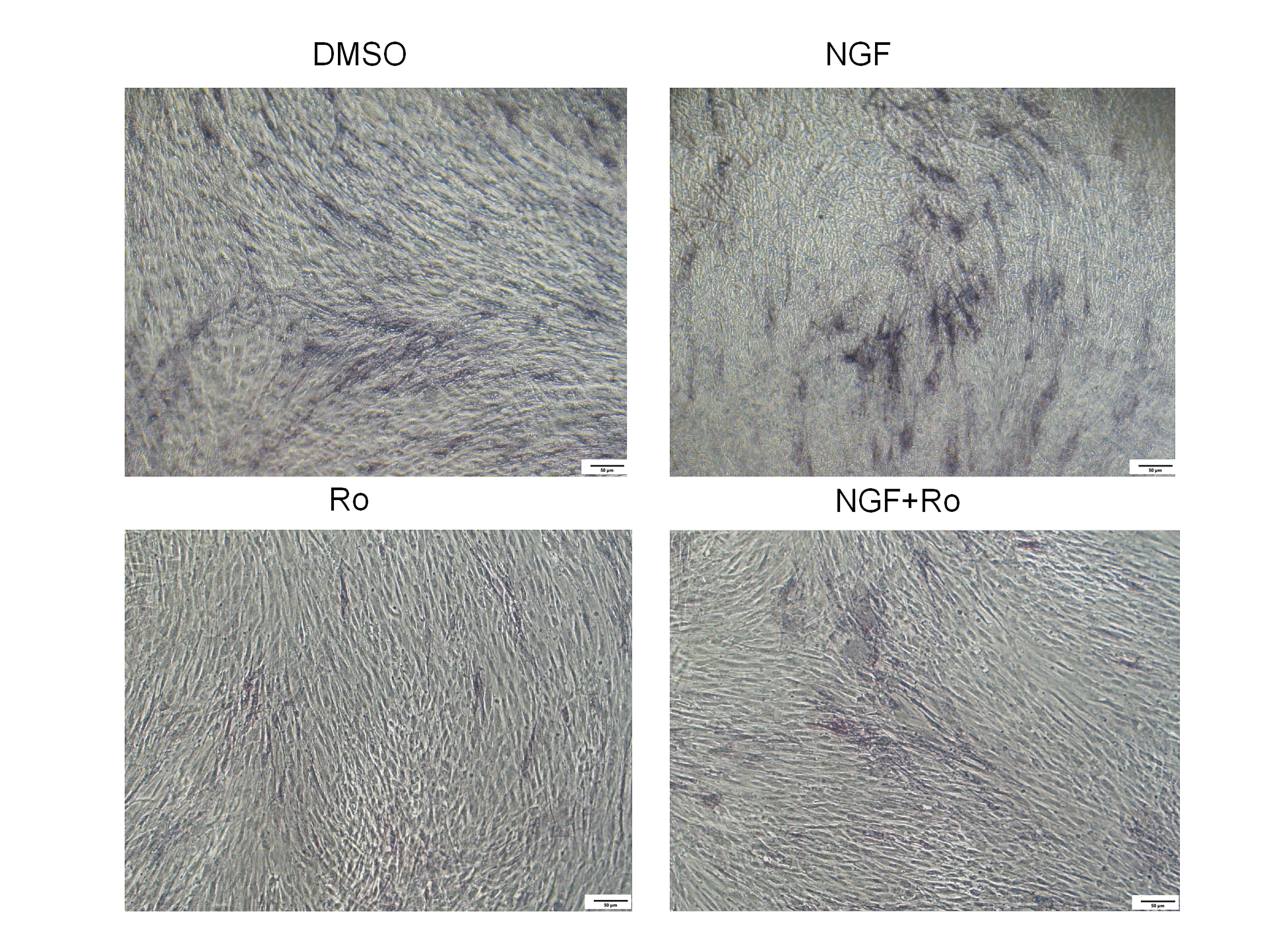
**

**Supplementary Figure 2** Treatment of DPSCs with Ro 08-2750 (10μM), a NGF inhibitor, could decrease the ALP staining (high magnification images). Scale bar = 50μm.
