# Supplementary material for: Nerve growth factor promote osteogenic differentiation of dental pulp stem cells through MEK/ERK signalling pathways
Source: J Cell Mol Med. 2024 Feb 9;28(4):e18143. doi: 10.1111/jcmm.18143 (PMC10853700; doi:10.1111/jcmm.18143)
Supplement: Supplementary file 3 — Table S1. [file JCMM-28-e18143-s003.docx]

**Supplementary Table 1. Dental Donor Information**

| **Sample number** | **Date** | **Sampling site** | **Method of sampling** | **Sample details** | **Note** |
| --- | --- | --- | --- | --- | --- |
| 1 | 2022/01/01 | 15 (Upper second premolar on the right maxillary) | orthodontic extraction | Patient's age 18, male, complete teeth, no caries, no inflammatory reaction, no pulp holes in teeth |  |
| 2 | 2022/01/01 | 38 (Left mandibular third molar tooth) | tooth extraction | Patient's age 20, male, complete teeth, no caries, no inflammatory reaction, no pulp holes in teeth |  |
| 3 | 2022/01/05 | 24 (First premolar on left maxillary) | orthodontic extraction | Patient's age 19, female, complete teeth, no caries, no inflammatory reaction, no pulp holes in teeth |  |
| 4 | 2022/01/05 | 35 (Left mandibular second premolar) | orthodontic extraction | Patient's age 19, male, complete teeth, no caries, no inflammatory reaction, no pulp holes in teeth |  |
| 5 | 2022/01/07 | 48 (Right mandibular third molar tooth | tooth extraction | Patient's age 18, female, no caries, no inflammatory reaction, no pulp holes in teeth | Teeth with minor fractures on the lingual surface do not affect the extraction of dental pulp stem cells |
| 6 | 2022/01/10 | 28(Left maxillary third molar) | tooth extraction | Patient's age 20, female, complete teeth, no caries, no inflammatory reaction, no pulp holes in teeth |  |
